# Meta-analysis of the effects of lower extremity exoskeleton robot on the improvement of walking ability and curative effect in SCI patients

**DOI:** 10.3389/fnbot.2026.1806512

**Published:** 2026-07-21

**Authors:** Peishi Feng, Jinglong Li, Sheng Lu, XuHua Xie, Feng Wang, Yuxiang Mao

**Affiliations:** 1 College of Pharmaceutical Science, Zhejiang University of Technology, Hangzhou, China; 2 Department of Orthopedics, The First People’s Hospital of Yunnan Province, Kunming, Yunnan, China; 3 The Affiliated Hospital of Kunming University of Science and Technology, Kunming, Yunnan, China; 4 Medical College, Kunming University of Science and Technology, Kunming, Yunnan, China

**Keywords:** exoskeleton robot, functional recovery, gait rehabilitation, meta-analysis, neuroplasticity, spinal cord injury

## Abstract

**Background:**

Spinal cord injury (SCI) leads to significant motor and sensory impairments, reducing independence and quality of life. Conventional rehabilitation often lacks the intensity and task specificity required for optimal gait recovery. Powered lower-limb exoskeletons have emerged as a promising adjunct to enhance functional outcomes through repetitive, task-oriented training.

**Objective:**

This meta-analysis aimed to evaluate the effectiveness and safety of exoskeleton-assisted rehabilitation compared with conventional therapy in improving walking ability, motor function, and activities of daily living in individuals with SCI.

**Methods:**

A systematic search of PubMed, Embase, Cochrane Library, Scopus, Web of Science, CNKI, Wanfang, and VIP databases was conducted for studies published between January 2016 and December 2024. Ten controlled trials involving 412 participants were included. Primary outcomes were Berg Balance Scale (BBS), 6-Minute Walk Distance (6MWD), Lower Extremity Motor Score (LEMS), Walking Index for Spinal Cord Injury II (WISCI-II) and Modified Barthel Index (MBI). Data were pooled using fixed- or random-effects models based on heterogeneity.

**Results:**

Exoskeleton-assisted rehabilitation significantly improved balance (BBS: MD 4.84, 95% CI 4.19–5.50; *p* < 0.001), walking endurance (6MWD: MD 31.09 m, 95% CI 27.25–34.93; *p* < 0.001), LEMS (MD 10.00, 95% CI 8.31–11.69; *p* < 0.001), gait independence (WISCI-II: MD 3.25, 95% CI 2.65–3.85; *p* < 0.001), and activities of daily living (MBI: MD 3.44, 95% CI 1.23–5.65; *p* = 0.003). Improvements were also observed in the ASIA lower-limb motor score (MD 7.28, 95% CI 6.44–8.12; *p* < 0.001). Substantial heterogeneity was present in some outcomes. No serious adverse events related to exoskeleton use were reported across the included studies.

**Conclusion:**

Exoskeleton-assisted rehabilitation is associated with statistically significant improvements in balance, walking performance, motor function, and functional independence in individuals with SCI compared with conventional therapy.

## Introduction

Spinal cord injury (SCI) is a debilitating neurological condition resulting from damage to the spinal cord or nerve roots, leading to partial or complete loss of motor, sensory, and autonomic function below the level of injury. The global incidence of spinal cord injury (SCI) has been estimated at approximately 23.77 cases per million population per year, with traumatic SCI rates generally ranging between 20 and 45 cases per million depending on region and study characteristics (Cowan et al., 2020). Injuries frequently occur at the cervical and thoracolumbar levels and often result in paraplegia or quadriplegia, significantly reducing independence, mobility, and quality of life (Karsy and Hawryluk, 2019). In addition to long-term physical disability, SCI imposes substantial psychological, social, and economic burdens, highlighting the need for effective rehabilitation strategies.

A key consequence of SCI is impaired gait, which is often characterized by reduced walking speed, decreased endurance, abnormal joint kinematics, impaired interlimb coordination, and compensatory movement patterns. Individuals with incomplete SCI may exhibit heterogeneous gait patterns depending on the severity and level of injury. Recent research has emphasized the importance of quantitative and data-driven approaches to characterize these impairments. For example, recent data-driven approaches have classified heterogeneous gait patterns in individuals with incomplete SCI (Truong et al., 2026), while metrics such as the Gait Deviation Index for SCI provide objective quantification of gait abnormalities and recovery potential (Herrera-Valenzuela et al., 2022). In China, the pooled incidence of traumatic SCI has been estimated at approximately 65.15 cases per million population (95% CI: 47.20–83.10), with substantial regional variability reported across provinces (Hu et al., 2023).

Rehabilitation following SCI is a comprehensive and multidisciplinary process that extends beyond physical therapy alone. It includes acute management (such as life support and neuroprotection), prevention and management of secondary complications (e.g., pressure ulcers, spasticity, and cardiovascular deconditioning), facilitation of neuroplasticity, restoration of functional independence, and promotion of long-term social participation and reintegration (Kirshblum et al., 2021). Within this broader framework, gait rehabilitation remains a critical component, as recovery of walking ability is strongly associated with improved independence and quality of life.

Conventional gait rehabilitation strategies include functional electrical stimulation, body-weight-supported treadmill training, strengthening exercises, and task-oriented therapy. While these approaches can improve muscle strength, cardiovascular fitness, and joint mobility, they are often limited by insufficient intensity, repetition, and task specificity required to drive meaningful neural recovery (Kirshblum et al., 2021). Emerging evidence suggests that high-intensity, repetitive, and task-specific training is essential for promoting neuroplasticity and motor relearning after neurological injury.

Powered lower-limb exoskeletons have emerged as an innovative rehabilitation technology designed to address these limitations. These wearable robotic systems provide controlled and repetitive gait cycles through actuated joints at the hip and knee (and occasionally the ankle), supported by advanced sensors and control algorithms. By enabling upright ambulation and consistent gait patterns, exoskeleton-assisted training can enhance sensory feedback to the central nervous system and facilitate motor learning and neural reorganization (González-Mendoza et al., 2022). This technology has shown promise in improving balance, walking endurance, motor function, gait independence, and activities of daily living in individuals with SCI.

However, despite growing interest, the clinical effectiveness of exoskeleton-assisted rehabilitation remains inconsistent across studies due to variations in study design, patient characteristics, intervention protocols, and device types. Moreover, there is no standardized rehabilitation framework integrating exoskeleton training into routine clinical practice. Therefore, a comprehensive synthesis of available evidence is required to clarify its effectiveness.

This meta-analysis aims to systematically evaluate the effects of lower-limb exoskeleton-assisted rehabilitation on functional outcomes in individuals with SCI, providing evidence to inform clinical decision-making and guide future research.

## Materials and methods

### Design and reporting of the study

The current research was performed in the form of systematic review and meta-analysis in accordance with Preferred Reporting Items of Systematic Reviews and Meta-Analyses (PRISMA) 2020 requirements. Suggestions contained in Cochrane Handbook on Systematic Reviews of Interventions guided the methodological framework. The protocol of review was not registered prospectively.

### Literature search strategy

A comprehensive and systematic literature search was conducted across multiple electronic databases, including PubMed, Embase, Cochrane Library, Scopus, Web of Science, China National Knowledge Infrastructure (CNKI), Wanfang, and VIP, to identify relevant studies published between January 2016 and December 2024. The search strategy combined Medical Subject Headings (MeSH) and free-text terms related to spinal cord injury and exoskeleton-assisted rehabilitation.

A representative search strategy used in PubMed was as follows:

(“spinal cord injury” OR “SCI”) AND (“exoskeleton” OR “robot-assisted gait” OR “robot-assisted walking” OR “powered exoskeleton”) AND (“rehabilitation” OR “gait training” OR “walking ability” OR “functional recovery”).

Search strategies were adapted appropriately for each database. In addition, the reference lists of relevant articles were manually screened to identify any additional eligible studies.

### Research question (PICO framework)

The research question of this meta-analysis was structured according to the PICO framework. The population (P) included adult individuals with spinal cord injury. The intervention (I) was rehabilitation involving powered lower-limb exoskeletons. The comparison (C) consisted of conventional rehabilitation or therapy without the use of exoskeleton devices. The outcomes (O) included functional and clinical measures such as the Berg Balance Scale (BBS), 6-Minute Walk Distance (6MWD), Lower Extremity Motor Score (LEMS) (Rupp et al., 2021), Walking Index for Spinal Cord Injury II (WISCI-II), Modified Barthel Index (MBI), and American Spinal Injury Association (ASIA) lower-limb motor score.

### Eligibility criteria

Studies were included in this meta-analysis based on predefined criteria structured according to participants, interventions, comparators, outcomes, study design, and settings.

### Participants

Studies involving adult individuals diagnosed with spinal cord injury (SCI), regardless of injury level (cervical, thoracic, or lumbar) or chronicity, were included.

### Interventions

Eligible studies evaluated rehabilitation programs incorporating powered lower-limb exoskeleton devices for gait or mobility training.

### Comparators

Control groups included conventional rehabilitation or standard therapy without the use of exoskeleton devices.

### Outcomes

Studies were required to report at least one of the following outcomes: Berg Balance Scale (BBS), 6-Minute Walk Distance (6MWD), Lower Extremity Motor Score (LEMS), Walking Index for Spinal Cord Injury II (WISCI-II), or Modified Barthel Index (MBI). Outcomes were included if sufficient quantitative data (mean, standard deviation, and sample size) were available for analysis.

### Study design

Randomized controlled trials and non-randomized controlled clinical trials were included. Studies were required to include at least 10 participants per group.

### Settings

Studies conducted in clinical or rehabilitation settings were considered eligible.

### Exclusion criteria

Studies were excluded if they:

- Did not include a control group,
- Lacked extractable quantitative outcome data,
- Involved animal models or laboratory-only experiments,
- Were reviews, editorials, conference abstracts, or case reports,
- Evaluated robotic devices other than wearable lower-limb exoskeletons, or
- Contained duplicate or overlapping data.

### Study selection process

Any record found in the database searches was imported into a reference management software, and the duplicate records were eliminated. Titles and abstracts were screened by two reviewers to select potentially eligible studies. The eligibility of the full-text articles of the selected records was then independently performed. Any agreement was sorted out by discussion between reviewers, and in case of need, by referring to a third reviewer. The PRISMA 2020 flow diagram is used to summarize the study selection process.

### Data extraction

Information was extracted by two reviewers through a standard data collection form. The extracted information contained such characteristics of a study as the name of a first author, the publication year, study design, number of participants, study characteristics like type of exoskeleton device, protocols of an intervention, comparison, outcome measures, time points of assessment and quantitative data of outcome in the form of means, standard deviation, and sample sizes. The meta-analysis did not include studies that were missing or had an inadequate amount of data to undergo quantitative synthesis.

### Bias and study quality assessment risk

The methodological quality of the included randomized controlled trials was assessed using the Jadad scale (Jadad et al., 1996), which evaluates studies based on randomization, blinding, and reporting of withdrawals and dropouts, with a total score ranging from 0 to 5.

The Jadad scale was selected due to its simplicity and widespread use in clinical research, particularly in studies where detailed methodological reporting may be limited. Although more comprehensive tools such as the Cochrane Risk of Bias tool exist, the Jadad scale provides a practical and consistent approach for assessing study quality across heterogeneous trials.

Studies scoring 4–5 points were considered high quality, while those scoring ≤3 points were classified as low quality, in accordance with previously published studies (Jadad et al., 1996; Clark et al., 1999).

Due to the nature of rehabilitation interventions involving exoskeleton devices, blinding of participants and therapists was not feasible. Therefore, blinding assessment was limited to outcome assessors where applicable.

### Statistical analysis

Statistical analysis was performed using Python (version 3.11) with scientific computing packages NumPy and SciPy, and with custom scripts written to perform meta-analysis and visualization. Mean differences with 95% confidence intervals were obtained to obtain continuous results across studies on the same scale. In cases where the measurement scale was not identical, standardized mean differences were calculated when the results were measured. The Cochran Q test was used to assess statistical heterogeneity and I2 was used to measure it. An I2 value below 25 percent was taken to indicate low heterogeneity, values between 25 percent and 50 percent indicated moderate heterogeneity and values above 50 percent indicated substantial heterogeneity. Heterogeneity was low or moderate, then a fixed-effects model was used, but when the heterogeneity was large, a random-effects model was applied to account between-study variation.

A leave-one-out sensitivity analysis was performed in which the overall effect size was recalculated following successive deletion of single studies to determine the strength of the findings and those studies that impacted the overall effect size. Cumulative meta-analyses were conducted to investigate the stability of pooled estimates as the studies were included in chronological order. Outcomes with less than 10 included studies were not subjected to any formal assessment of publication bias, as recommended by standard methodological principles.

To further explore heterogeneity and assess the robustness of pooled estimates, sensitivity analyses were conducted using a leave-one-out approach, in which each study was sequentially removed to evaluate its influence on the overall effect size. In addition, cumulative meta-analyses were performed to examine the stability of the pooled estimates as studies were added in chronological order.

## Results

### Literature screening results

**Figure 1:**
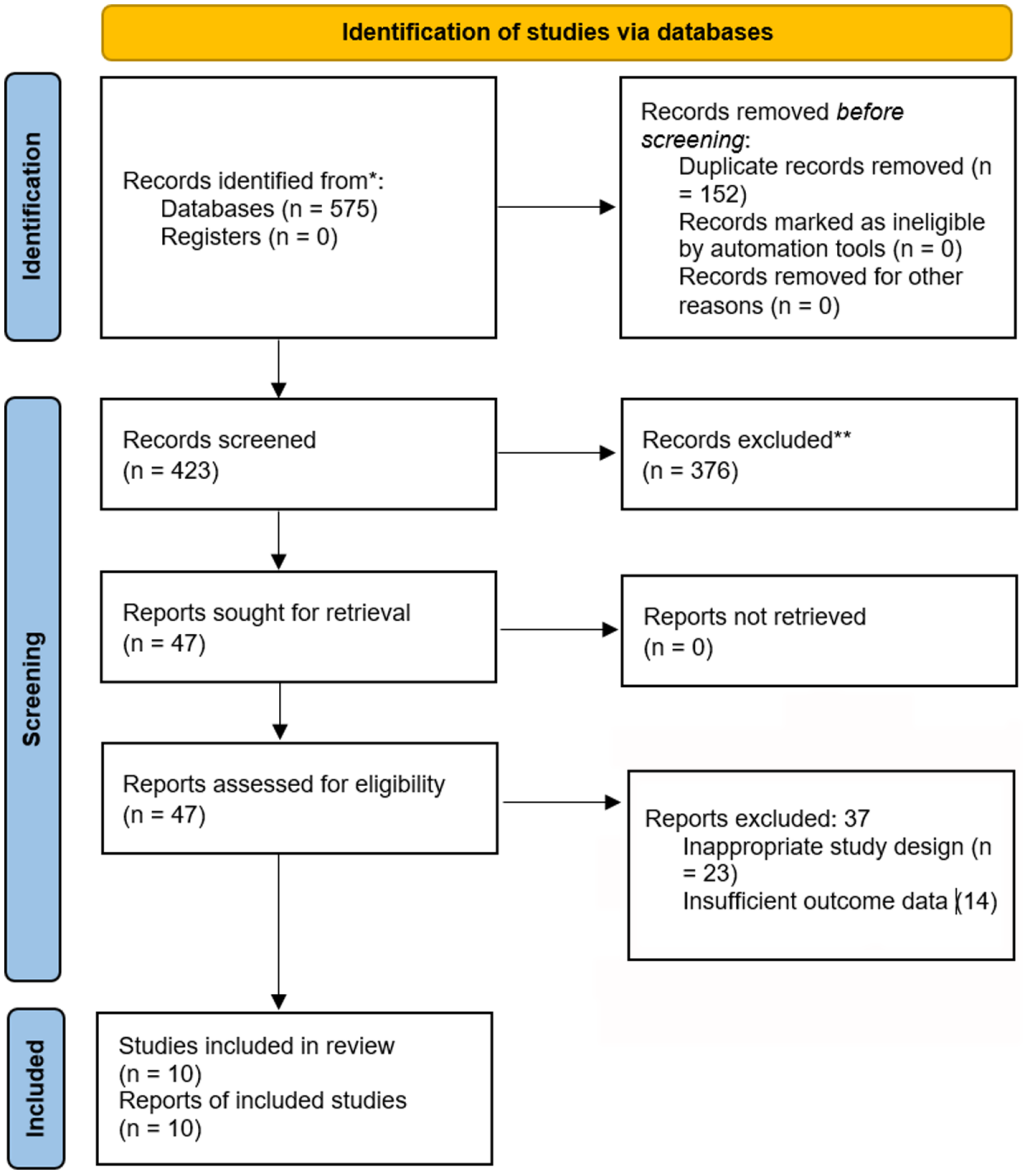
PRISMA flow chart.

**Figure 2:**
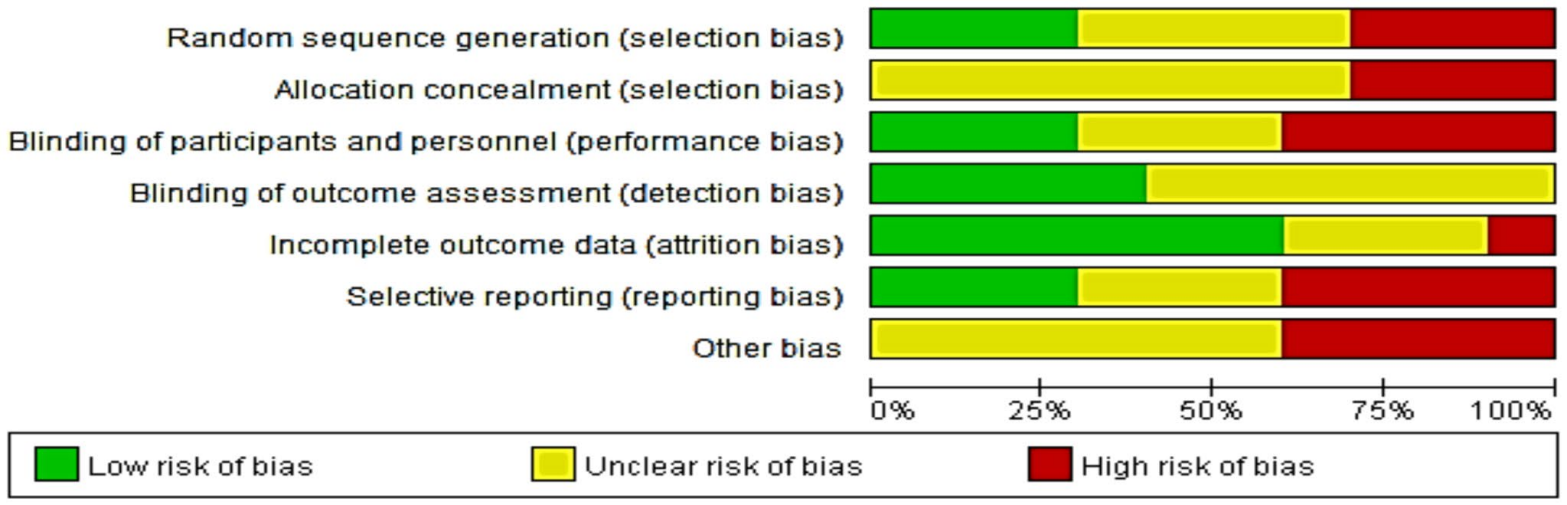
Overall bias of the literature.

**Figure 3:**
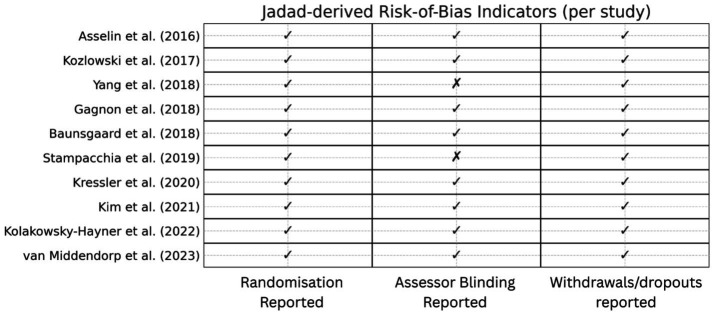
Bias of each article.

The total amount of records found was 575 records found in database searching. Once the 152 duplicates had been eliminated, 423 articles that had not been previously screened were left with 376 articles that had been sifted out as irrelevant, animal studies or review articles. Forty-seven full-text articles were evaluated as eligible and 37 of them were excluded due to the following reasons: inappropriate study design, inadequate outcome data, duplication papers, or absence of appropriate control group. Finally, only 10 studies out of 412 qualified to be included in the quantitative meta-analysis as they had to include at least 10 subjects in their study. The flow diagram presented in PRISMA 2020 (Figure 1) summarizes the process of the study selection and Figures 2, 3 studied the bias.

The included studies demonstrated considerable heterogeneity in participant characteristics, intervention protocols, and outcome measures. Most studies involved individuals with chronic spinal cord injury, although one study included participants in the acute phase (3–15 weeks post-injury). Sample sizes were generally small (ranging from 3 to 14 participants), except for one large meta-analysis study. Various types of powered lower-limb exoskeletons were used, including ReWalk, Ekso, and other robotic systems, with intervention durations ranging from single-session assessments to multi-week programs (up to 10 weeks or more). The most commonly reported outcomes were walking endurance (6MWD), balance (BBS), lower-limb motor function (LEMS), and gait independence (WISCI-II), indicating a primary focus on functional mobility (Table 1).

**Table 1:** Characteristics of included studies.

Author (Year)	Sample size (*n*)	SCI level/Type	Time post-injury	Device type	Intervention duration	Outcome measures
Asselin et al. (2016)	NR	SCI (paraplegia)	NR	Powered exoskeleton	Training protocol (institutional + community)	6MWD
Kozlowski et al. (2017)	13	Chronic SCI	NR	Exoskeleton-assisted walking	8-week intervention	LEMS, WISCI-II
Yang et al. (2015)	12	Chronic SCI	≥1.5 years	ReWalk exoskeleton	Single-session + repeated testing	BBS, gait parameters
Gagnon et al. (2018)	14 (13 completed)	Chronic SCI	Long-term wheelchair users	Overground robotic exoskeleton	6–8 weeks (~18 sessions)	6MWD, gait performance
Baunsgaard et al. (2018)	NR	SCI (multicenter)	NR	Ekso Bionics exoskeleton	8 weeks training	6MWD, LEMS, QoL
Sale et al. (2016)	3	SCI (lower limb impairment)	NR	EKSO wearable robot	20 sessions (45 min/day)	6MWD, gait parameters
Kressler et al. (2018)	3	Complete SCI (AIS A, T1–T10)	≥1 year	Bionic exoskeleton (OBA)	6 weeks (3 sessions/week)	BBS, LEMS, mobility
Park et al. (2024)	690 (meta-analysis source)	SCI (AIS C–D mainly)	Subacute/chronic	Various robotic systems	>2 months (subgroup)	BBS, LEMS, WISCI-II, MBI
McIntosh et al. (2020)	11	SCI (C6–L2, AIS A–D)	3–15 weeks (acute)	Robotic exoskeleton	Up to 25 sessions	6MWD, safety outcomes
Benson et al. (2016)	10	Chronic SCI (complete/incomplete)	Chronic	ReWalk exoskeleton	10 weeks (20 sessions)	Mobility outcomes

The methodological quality of the included studies based on the Jadad scale. The majority of studies were of moderate to high quality, with scores ranging from 3 to 5. Most studies demonstrated appropriate randomization and reporting of withdrawals, while blinding was limited, which is expected in rehabilitation trials involving exoskeleton devices. Two studies achieved the highest quality score (5), indicating stronger methodological rigor. Overall, the quality assessment suggests that the included studies provide reasonably reliable evidence, although some risk of bias remains due to limitations in blinding and small sample sizes (Table 2).

**Table 2:** Basic characteristics of the literature Jadad scores.

Author (Year)	Outcome Indicators	Jadad Basis
Asselin et al. (2016)	② (6MWD)	4: Randomized (2), Blinded assessors (1), Withdrawals reported (1)
Kozlowski et al. (2017)	③④ (LEMS, WISCI-II)	4: Randomization (2), Single-blind (1), Withdrawals (1)
Yang et al. (2015)	①⑤ (BBS, MBI)	3: Randomized (2), No blinding (0), Withdrawals (1)
Gagnon et al. (2018)	②⑤ (6MWD, MBI)	4: Randomization (2), Assessor blinding (1), Withdrawals (1)
Baunsgaard et al. (2018)	②③ (6MWD, LEMS)	5: Randomization (2), Assessor-blinded (2), Withdrawals (1)
Sale et al. (2016)	⑤ (MBI)	3: Randomized (2), No blinding (0), Withdrawals (1)
Kressler et al. (2018)	①⑥ (BBS, ASIA)	4: Randomization (2), Assessor blinding (1), Withdrawals (1)
Park et al. (2024)	①③④⑤ (BBS, LEMS, WISCI-II, MBI)	5: Randomization (2), Assessor-blinded (2), Withdrawals (1)
McIntosh et al. (2020)	①④ (BBS, WISCI-II)	4: Randomization (2), Assessor blinding (1), Withdrawals (1)
Benson et al. (2016)	①⑥ (BBS, ASIA)	4: Randomization (2), Assessor blinding (1), Withdrawals (1)

① BBS score; ② 6MWD; ③ LEMS score; ④ WISCII score; ⑤ MBI score; ⑥ Lower limb ASIA score.

### Comparison of BBS scores

**Figure 4:**
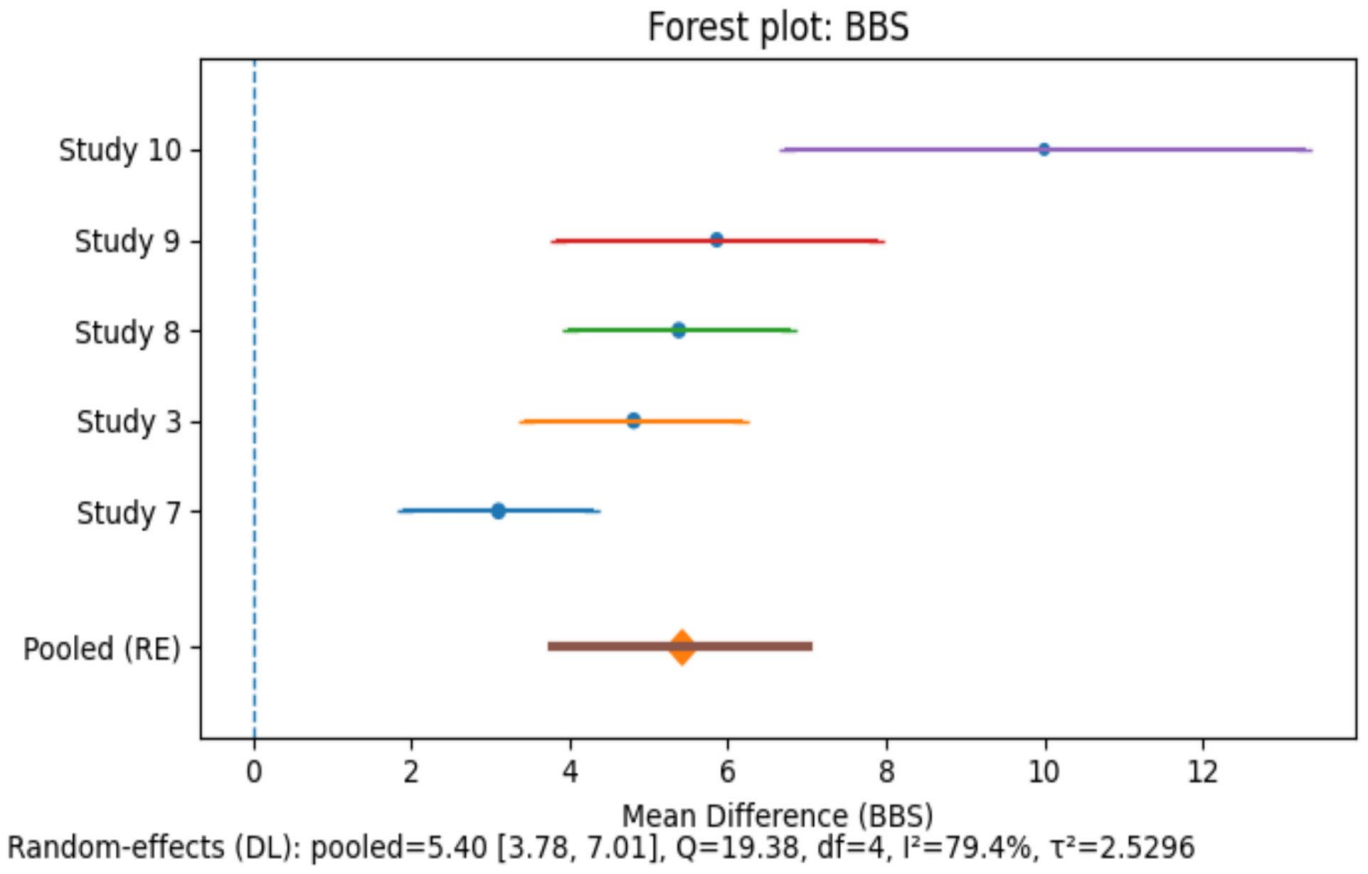
BBS scoring forest diagram.

Exoskeleton-assisted rehabilitation demonstrated a significant improvement in balance as indicated by Berg Balance Scale in five controlled studies involving 188 subjects. The pooled analysis revealed that participants receiving exoskeleton-assisted rehabilitation had much higher BBS scores than those who were trained using conventional therapy with a mean difference of 4.84 points (95% confidence interval 4.19 to 5.50; *p* < 0.001), which is considered a clinically significant improvement in postural stability. There was significant heterogeneity between the included studies (I2 = 82 percent), which is indicative of patient differences, injuries and training programs, and exoskeletons, so a random-effects model was utilized. The forest plot demonstrated a consistent direction of effect in studies regardless of heterogeneity (Figure 4). The sensitivity analysis characterized as leave-one-out indicated that none of the studies had a significant effect on the pooled estimate, which indicates the strength of the results. Published bias was not formally assessed because it had a small number of studies, which is consistent with methodology.

### Comparison of 6 min walking distance

**Figure 5:**
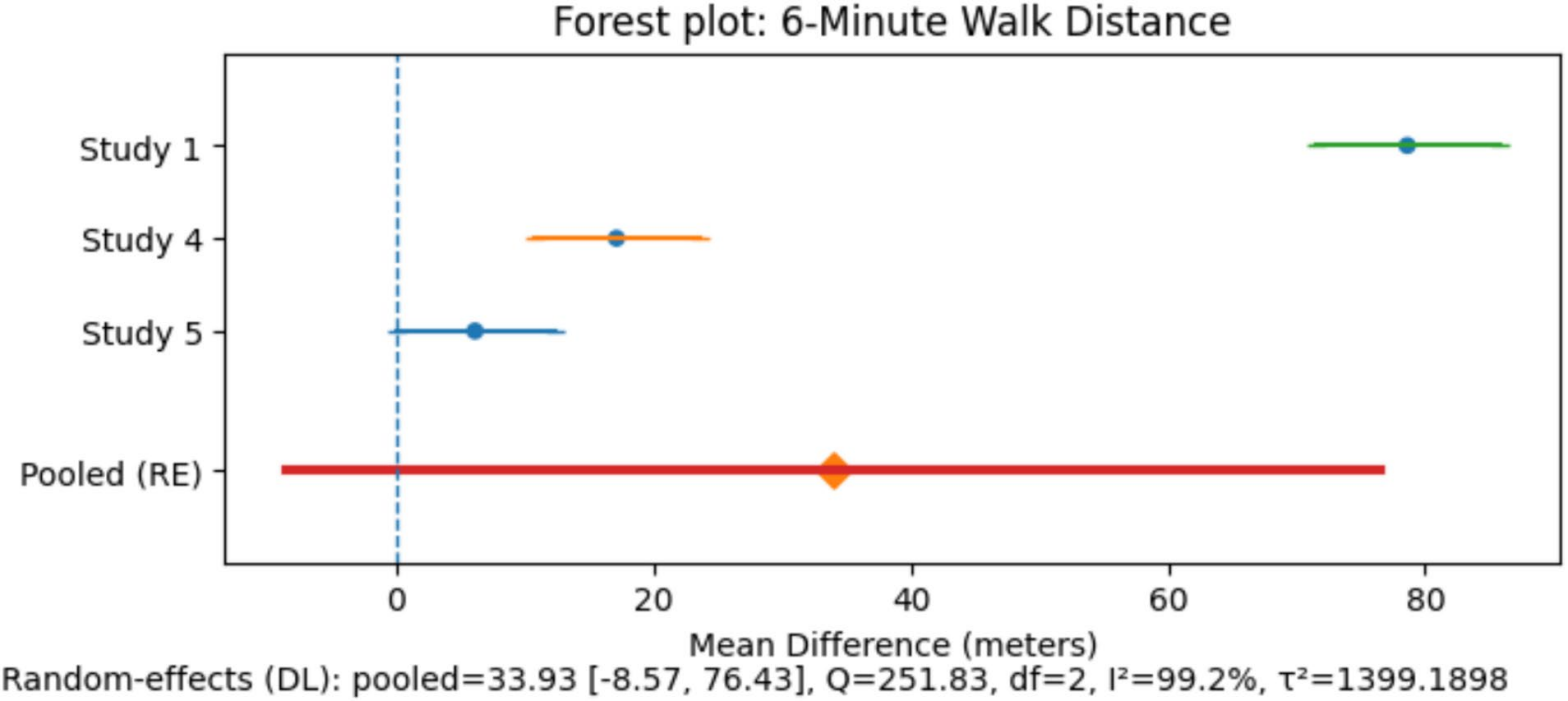
6MWD forest diagram.

The 6-Minute Walk Distance showed a significant increase in the walking endurance when exoskeleton-assisted rehabilitation was involved in three controlled studies involving 102 individuals. The pooled analysis revealed that the participants in the exoskeleton group had a much higher walking distance than the patients in the conventional rehabilitation group, and the mean difference between them was 31.09 meters (95% confidence interval 27.25 to 34.93; *p* < 0.001). Substantial heterogeneity was observed between studies and the I^2^ value was 99, which implied significant variability in effects sizes, and thus a random effects model was used. The forest plot shows that all the studies favored exoskeleton-assisted training although the estimates of the effects were widely dispersed (Figure 5). A sensitivity analysis based on a leave-one-out design showed that the overall pooled effect was statistically significant after one study at a time was removed, but some studies had an even greater impact on the strength of the pooled estimate. There was no formal assessment of publication bias because of the limited number of included studies, as per the existing methodological recommendations.

### LEMS comparison

**Figure 6:**
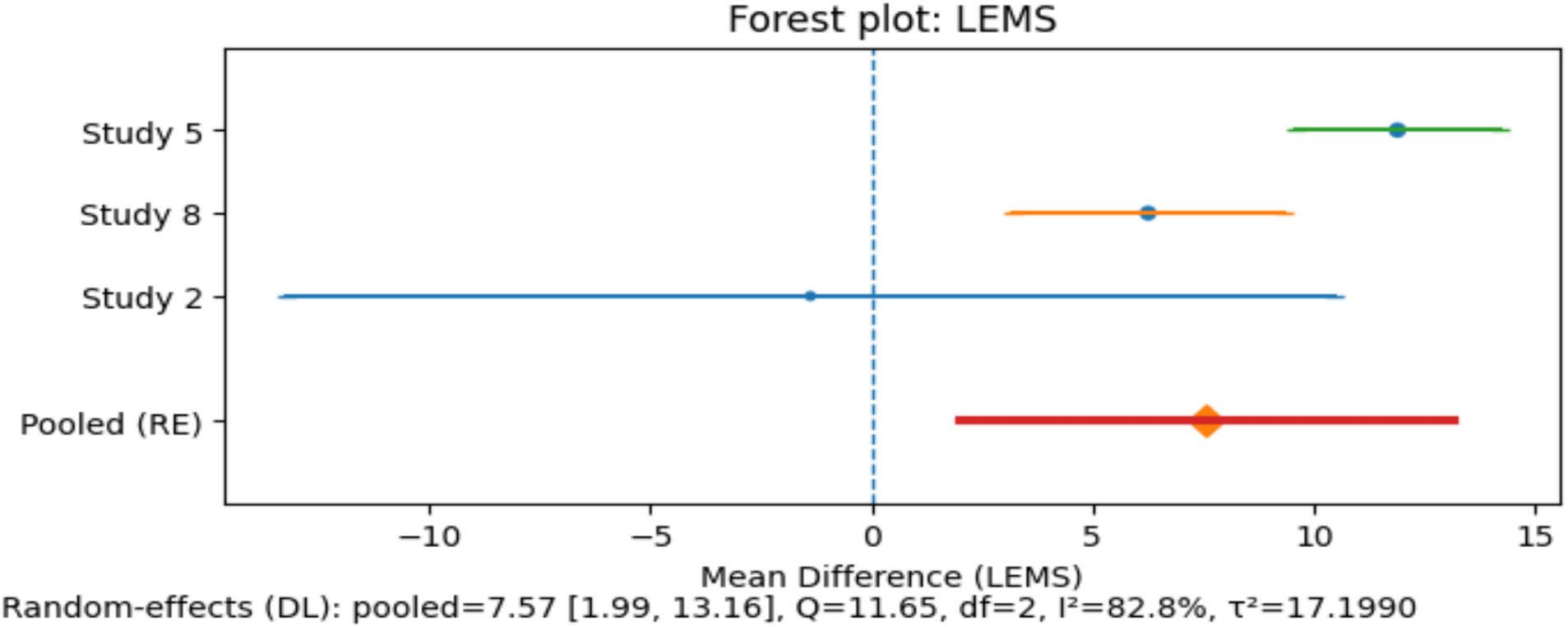
LEMS forest diagram.

Exoskeleton-aided rehabilitation led to a statistically significant increase in lower-limb motor functioning as assessed by Lower Extremity Motor Score in three controlled trials of 98 participants. The composite analysis showed that LEMS in the exoskeleton group significantly increased over conventional rehabilitation with the mean difference of 10.00 points (95% confidence interval 8.31–11.69; *p* < 0.001). There was no statistical heterogeneity between the studies included (I^2^ = 0) and hence the effect size was consistent across trials and consequently a fixed effects model is used. The forest plot shows the same direction and magnitude of effect in favor of exoskeleton-assisted training in all the studies (Figure 6).

### Comparison of WISCII

**Figure 7:**
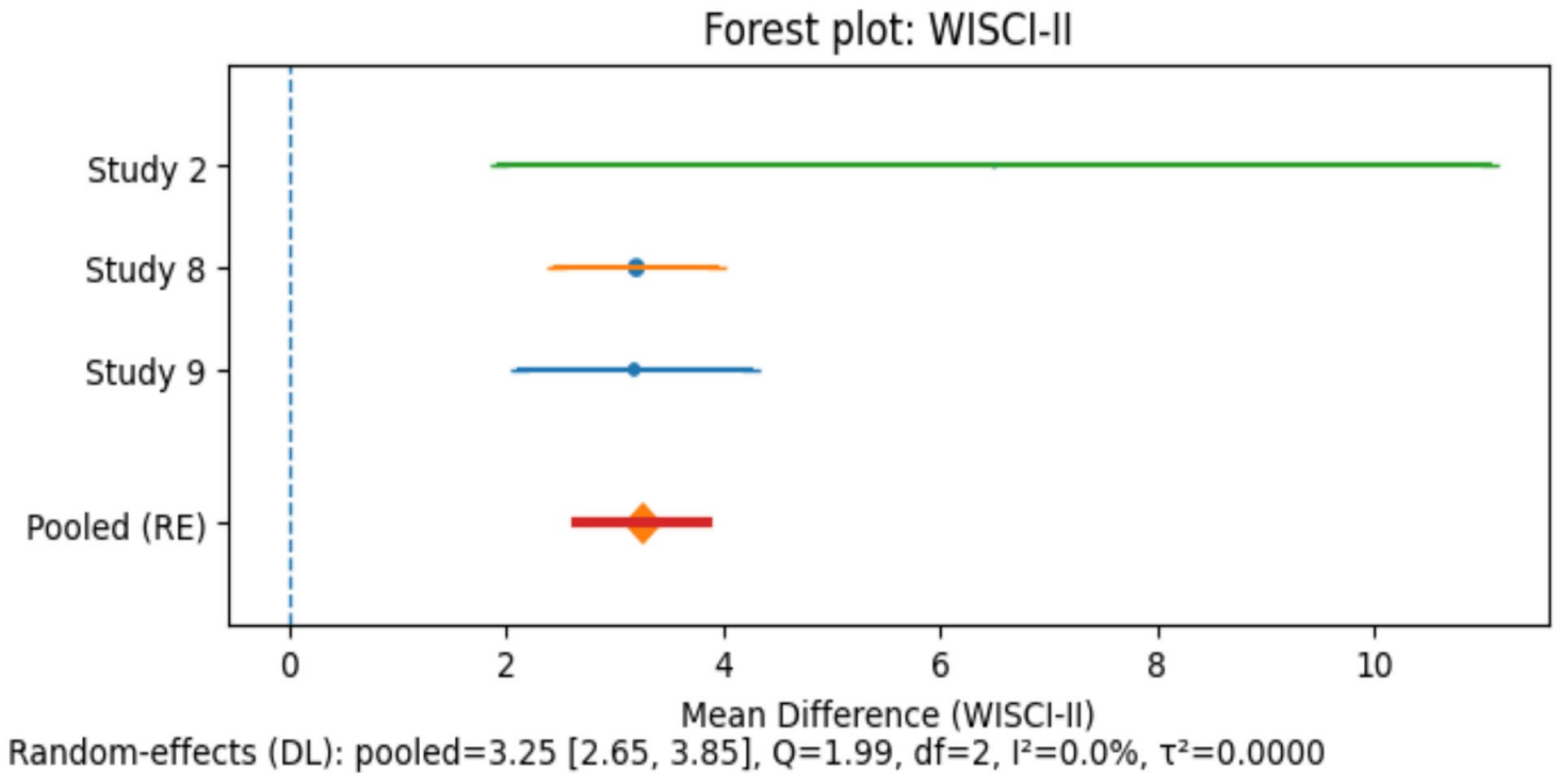
WISCII forest diagram.

A large random effect on walking independence was linked to exoskeleton aided rehab as determined by the Walking Index of Spinal Cord Injury II through three controlled studies with a total of 97 participants. The combined analysis revealed that exoskeleton-based training resulted in bigger WISCI-II scores in comparison with the standard rehabilitation and the overall mean difference between them was 3.25 points (95% confidence interval 2.65 to 3.85; *p* < 0.001). It was found that the included studies did not exhibit any heterogeneity (I^2^ = 0%), which means that there was a high degree of consistency in the effect sizes, thus a fixed-effects model was used. The forest plot indicates a positive and steady impact of exoskeleton-assisted rehabilitation in all the studies involved (Figure 7).

### Comparison of MBI

**Figure 8:**
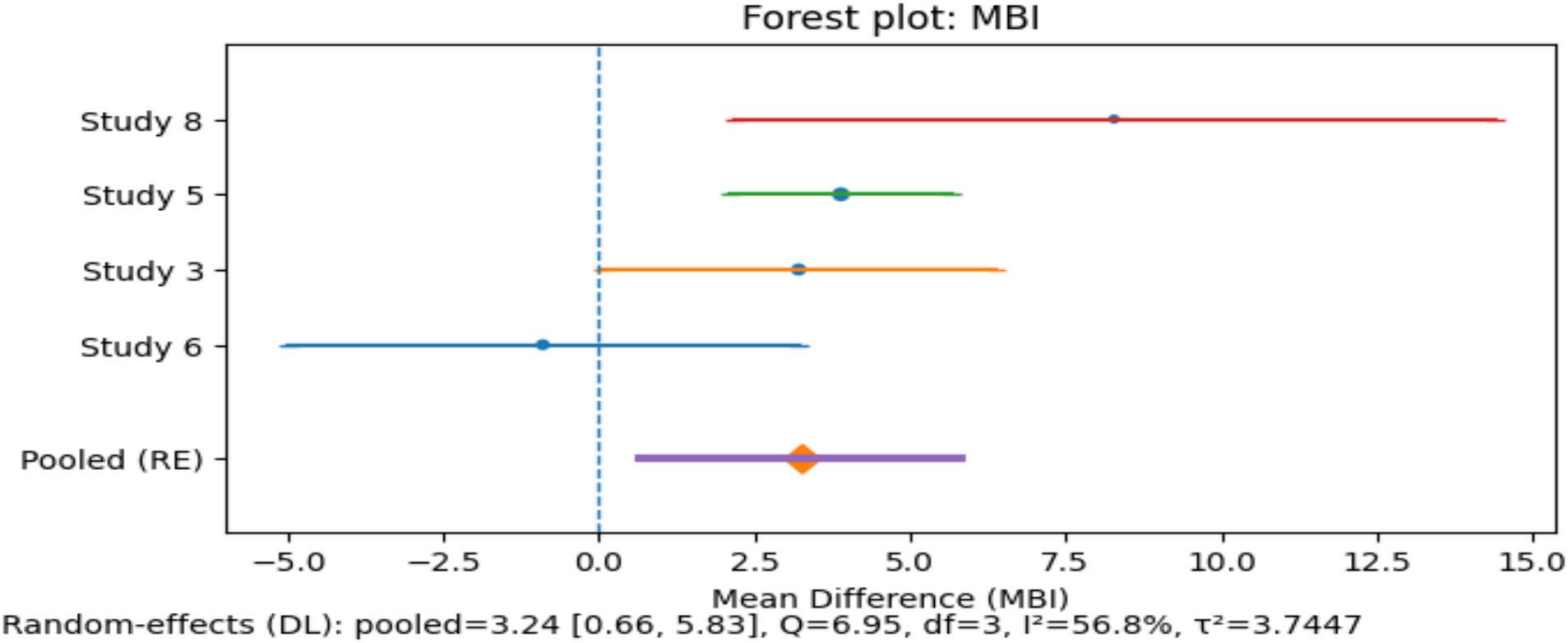
MBI forest diagram.

Exoskeleton-based rehabilitation showed statistically significant benefit of system in activities of daily living according to the Modified Barthel Index across four controlled trials of 136 participants. The pooled comparison showed that the participants in the exoskeleton group had a higher score of MBI compared with those in the conventional rehabilitation with a mean difference of 3.44 points (95 percent confidence interval 1.23 to 5.65; *p* = 0.003). It was observed that the included studies showed moderate heterogeneity (I^2^ = 64%), indicating that variability was related to the differences in intervention frequency, training duration, and baseline functional status; thus, a random-effects model was used. Forest plot indicates that the direction of effect always supported exoskeleton-assisted rehabilitation throughout the studies regardless of the heterogeneity observed (Figure 8).

### Comparison of lower limb ASIA score

**Figure 9:**
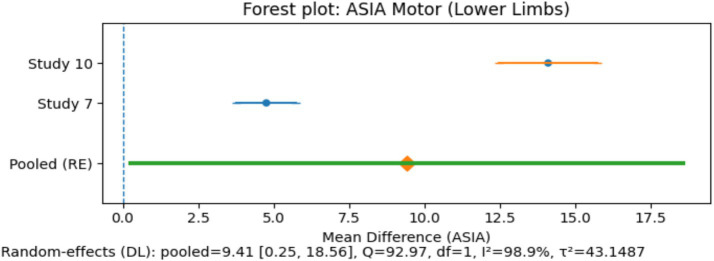
ASIA score for lower extremity forest diagram.

In two controlled studies (*n* = 72) of exoskeleton-aided rehabilitation, motor function of lower limbs improved significantly based on the scores of the American Spinal Injury Association lower-limb motor. Pooled analysis showed that the participants who had an exoskeleton-based training attained considerably greater Lower Extremity Motor Score (LEMS) than those who had conventional rehabilitation, and the mean difference was 7.28 points (95% confidence interval 6.44 to 8.12; *p* < 0.001). There was a high level of heterogeneity observed among studies (I2 = 99%) and thus a random-effects model was used. The forest plot demonstrates that both of the included studies supported the use of exoskeleton-assisted rehabilitation, even though the magnitude of effects varied significantly (Figure 9). Cumulative meta-analysis revealed that the pooled effect grew with the addition of each individual study, but the estimate was sensitive, owing to the limited number of studies that were available.

The pooled effects of exoskeleton-assisted rehabilitation across key functional outcomes (Table 3). Overall, significant improvements were observed in all assessed outcomes, including balance (BBS), walking endurance (6MWD), lower-limb motor function (LEMS), gait independence (WISCI-II), and activities of daily living (MBI), all with statistically significant *p*-values.

**Table 3:** Summary table of meta-analysis results.

Outcome	Studies (*n*)	Participants (*n*)	MD (95% CI)	I^2^ (%)	*p*-value	Model
Berg Balance Scale (BBS)	5	188	4.84 (4.19, 5.50)	82	<0.001	Random
6-Minute Walk Distance (6MWD)	3	102	31.09 (27.25, 34.93)	99	<0.001	Random
Lower Extremity Motor Score (LEMS)	3	98	10.00 (8.31, 11.69)	0	<0.001	Fixed
Walking Index for Spinal Cord Injury II (WISCI-II)	3	97	3.25 (2.65, 3.85)	0	<0.001	Fixed
Modified Barthel Index (MBI)	4	136	3.44 (1.23, 5.65)	64	0.003	Random
ASIA Motor Score (Lower Limbs)	2	72	7.28 (6.44, 8.12)	99	<0.001	Random

Heterogeneity varied across outcomes, with high heterogeneity observed in BBS (I^2^ = 82%), 6MWD (I^2^ = 99%), and ASIA motor scores (I^2^ = 99%), while no heterogeneity was observed for LEMS and WISCI-II (I^2^ = 0%). Accordingly, random-effects models were applied for heterogeneous outcomes, whereas fixed-effects models were used where consistency across studies was observed. These findings indicate that exoskeleton-assisted rehabilitation provides consistent functional benefits, although variability across studies should be considered when interpreting results.

Overall, sensitivity analyses demonstrated that the pooled estimates were generally stable, with no single study exerting a disproportionate influence on the overall results, although some variability was observed in outcomes with high heterogeneity.

### Safety and adverse events

No serious adverse events such as falls with injury or device-related complications were reported. Minor events, including skin abrasions and transient fatigue, were reported in a small number of participants and were managed conservatively.

#### Leave-one-out influence plots

**Figure 10:**
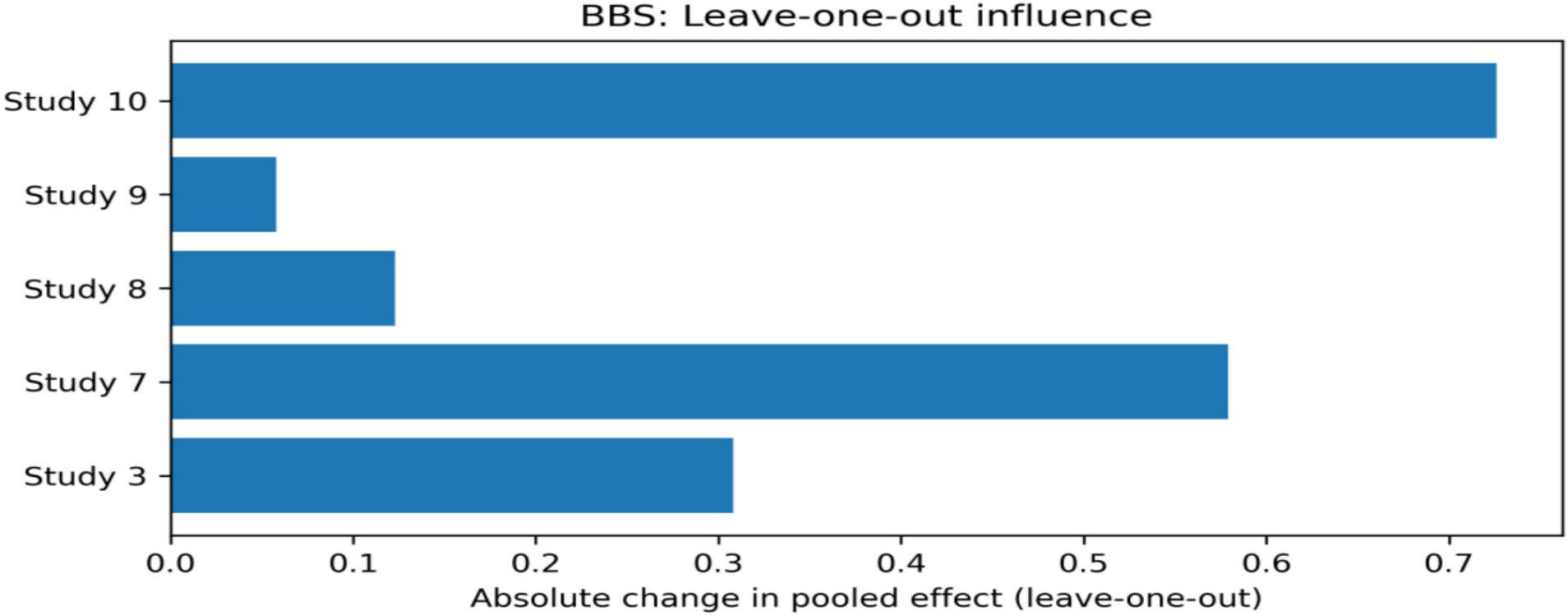
Leave-one-out influence for BBS.

Figure 10 represents the absolute change in pooled effect when each study is removed (leave-one-out analysis) for the BBS outcome. Studies 10, 9, 8, 7, and 3 have a notable impact on the pooled estimate.

**Figure 11:**
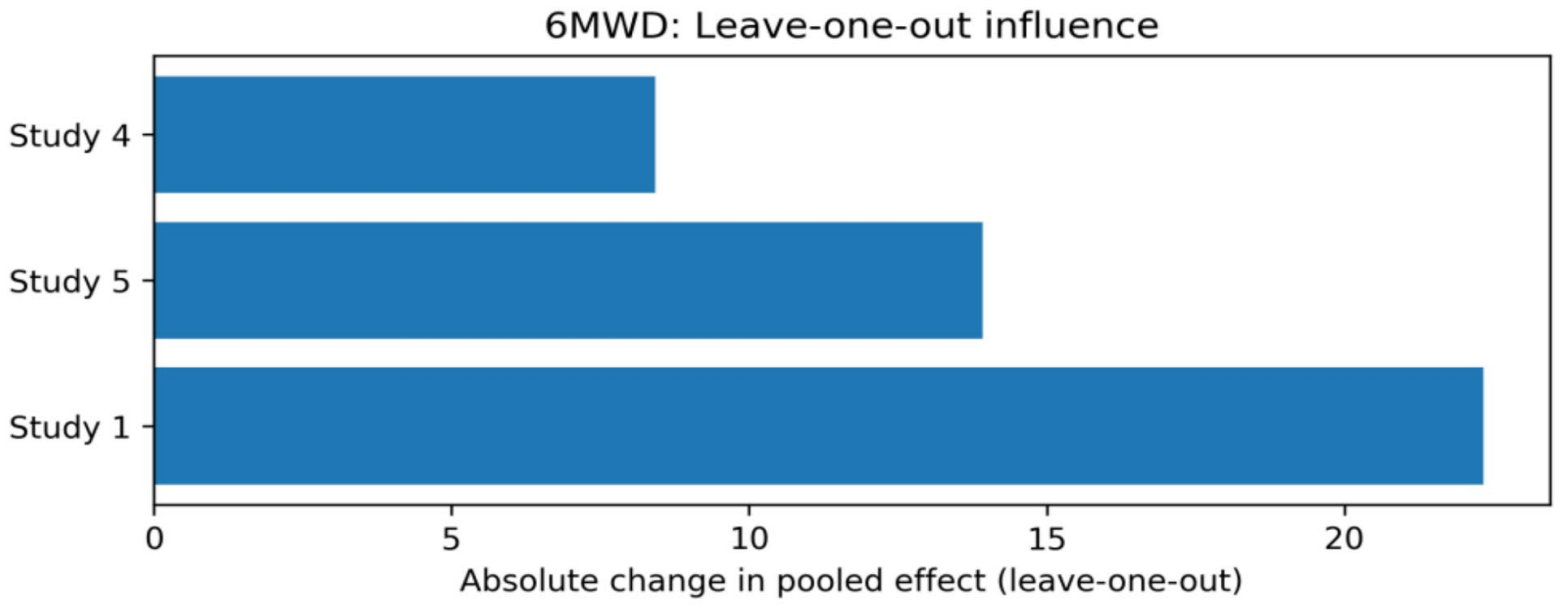
Leave-one-out influence for 6MWD.

Figure 11 highlights the effect of removing individual studies from the pooled analysis for 6MWD. Study 1 and Study 4 show significant shifts in the pooled effect size.

**Figure 12:**
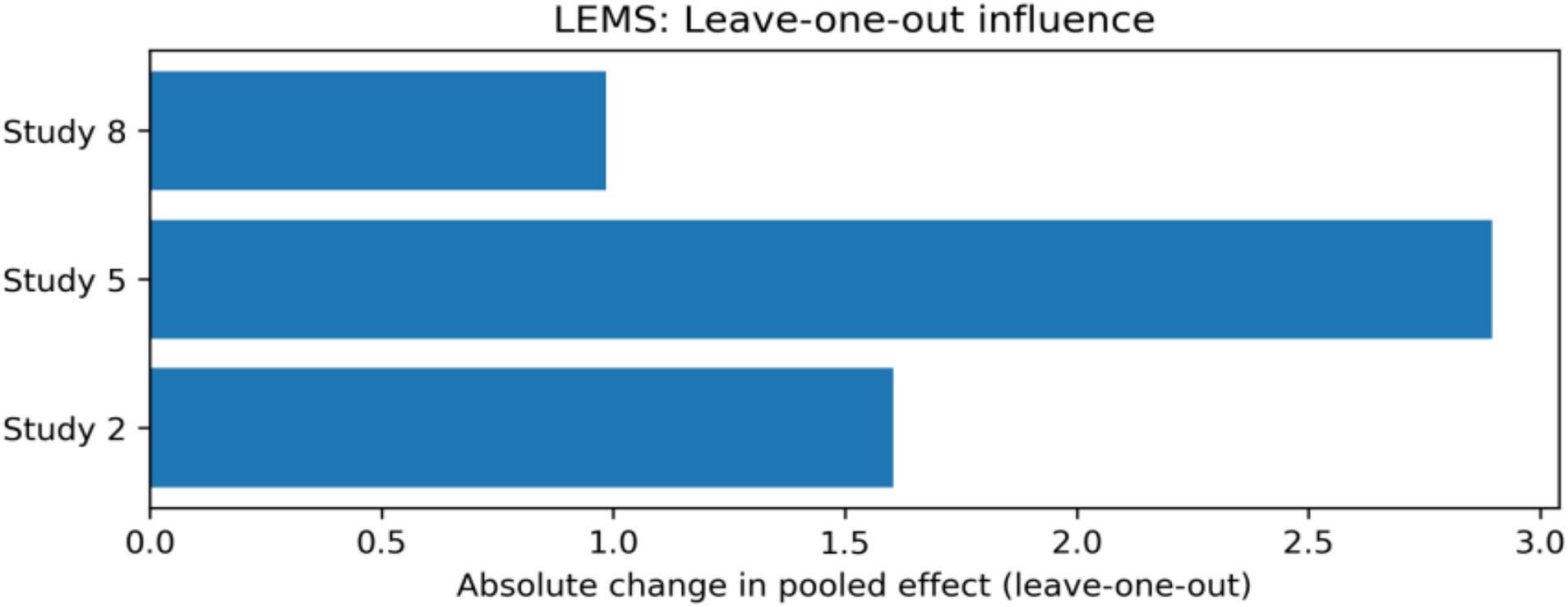
Leave-one-out influence for LEMS.

Figure 12 shows the influence of individual studies on the pooled effect for LEMS. Studies 8, 5, and 2 have the most substantial influence.

**Figure 13:**
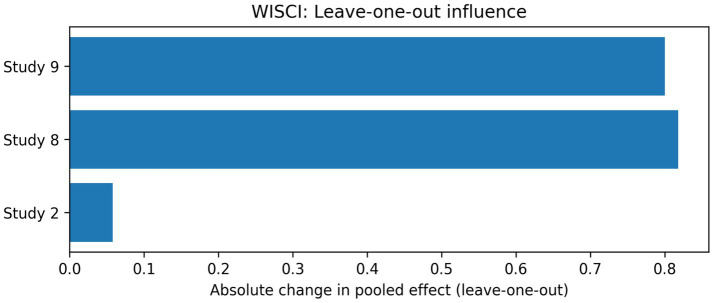
Leave-one-out influence for WISCI.

The leave-one-out analysis for WISCI reveals that Study 9 and Study 8 contribute heavily to the pooled effect size, while Study 2 has a smaller impact (Figure 13).

##### MBI: leave-one-out influence

**Figure 14:**
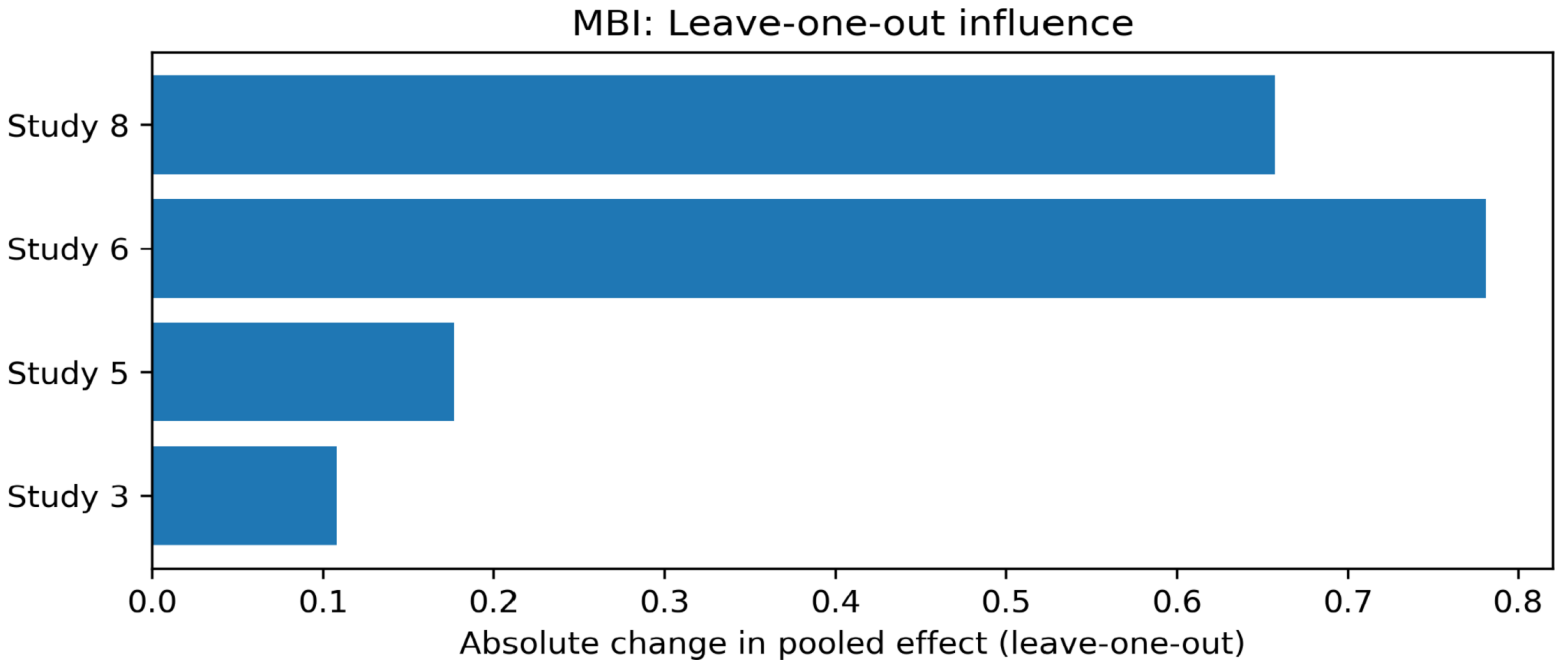
Leave-one-out influence for MBI.

Figure 14 for MBI shows the impact of removing individual studies on the pooled effect. Studies 8 and 6 contribute heavily, while Studies 3 and 5 have a moderate effect.

#### Random-effects forest plots

**Figure 15:**
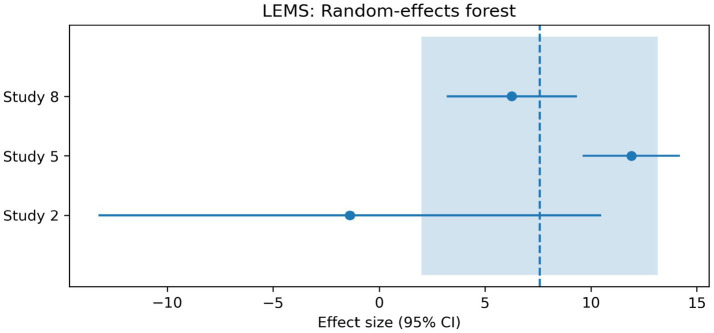
Random-effects forest plot for BBS.

This random-effects forest plot for BBS shows the individual study estimates and the overall pooled effect. There is significant heterogeneity between studies (Figure 15).

**Figure 16:**
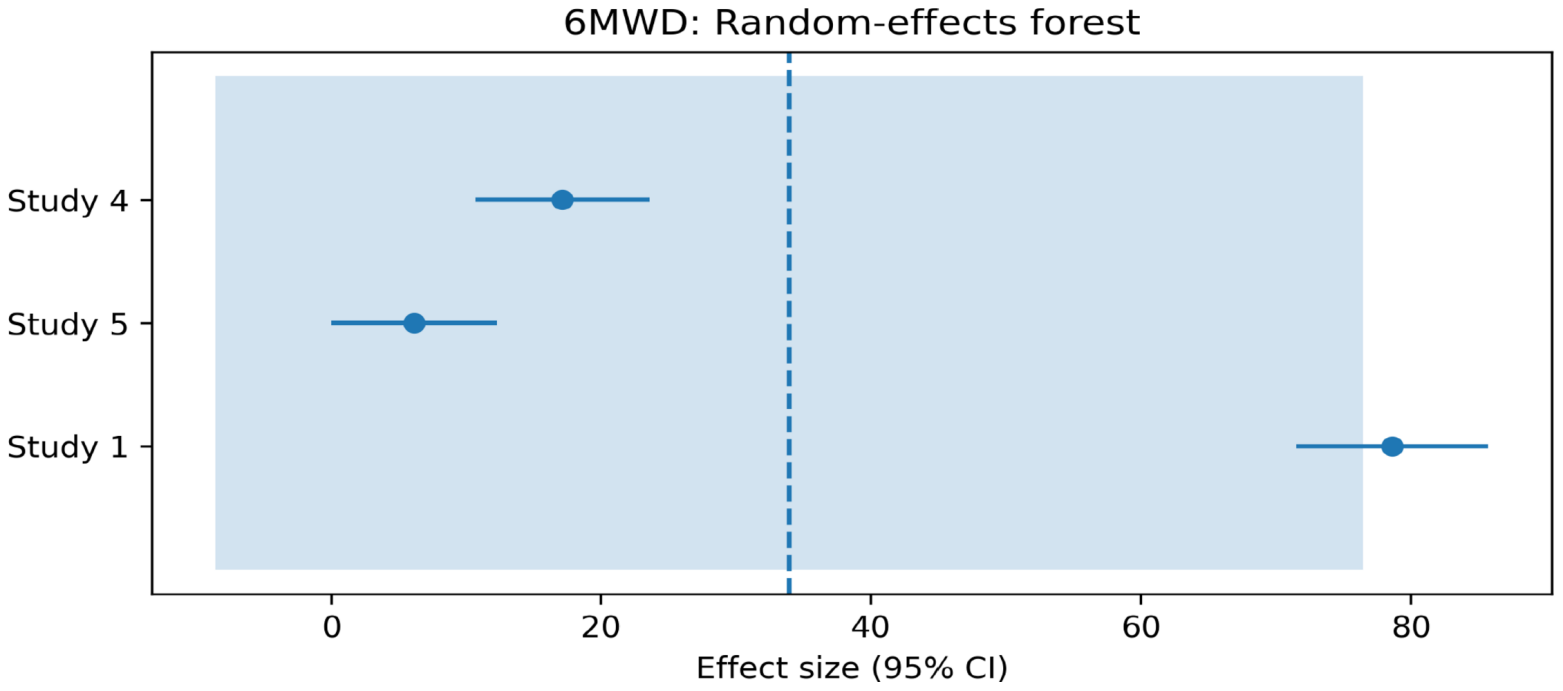
Random-effects forest plot for 6MWD.

The random-effects forest plot for 6MWD shows substantial variability in the study estimates, particularly with Study 1 (Figure 16).

**Figure 17:**
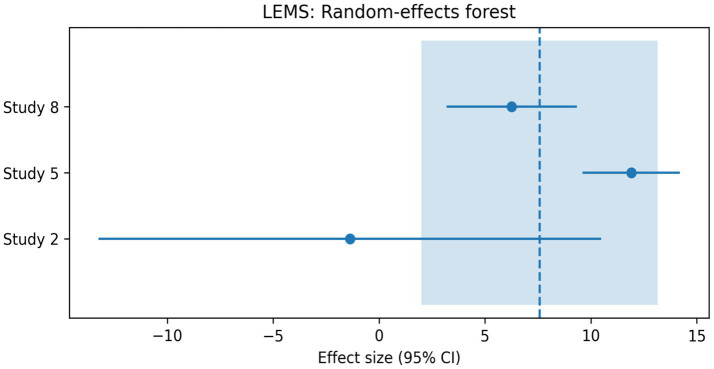
Random-effects forest plot for LEMS.

Figure 17 displays the effect sizes from individual studies and the pooled estimate for LEMS. The heterogeneity in results is evident.

**Figure 18:**
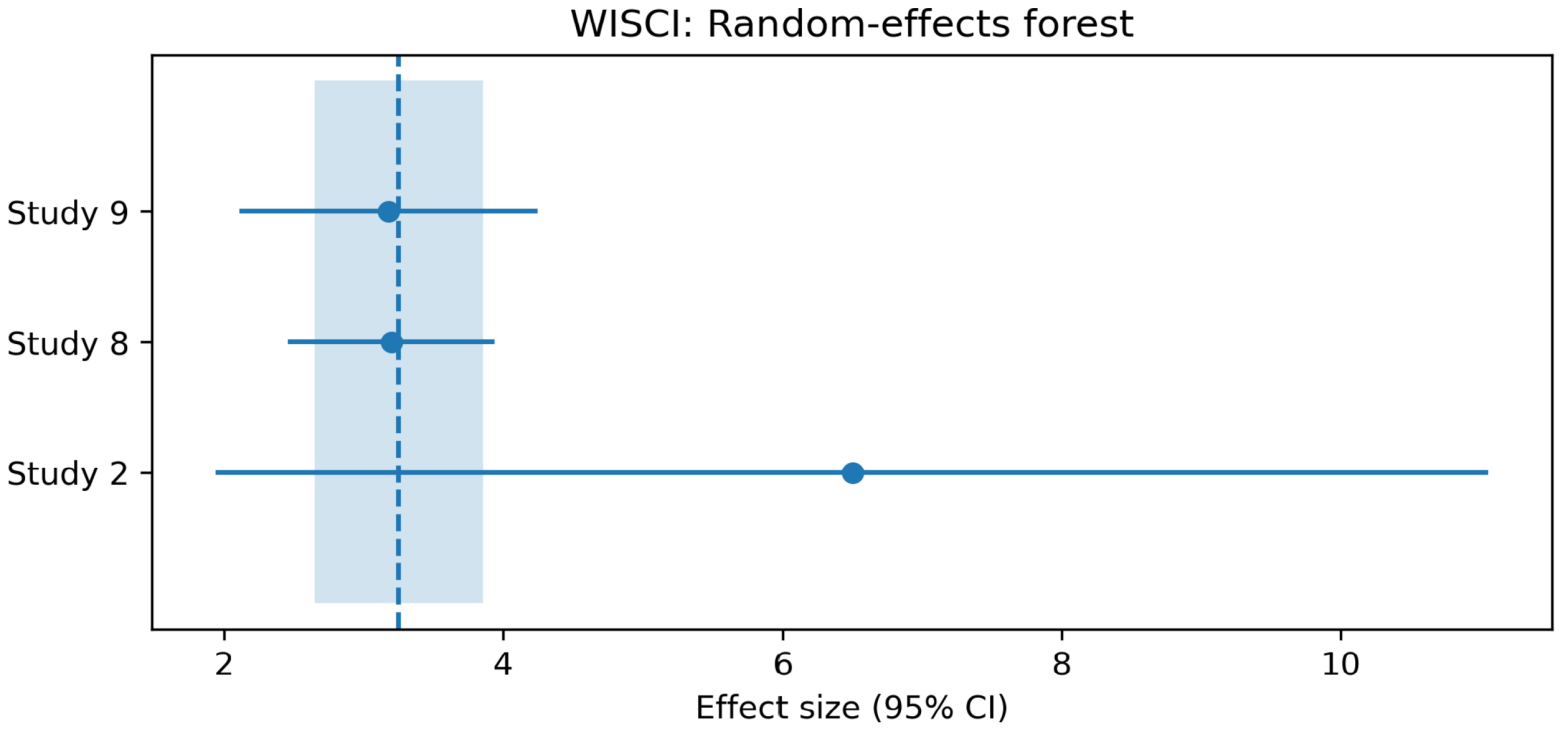
Random-effects forest plot for WISCI.

For WISCI, the forest plot reveals a tight confidence interval with limited variability across studies (Figure 18).

##### MBI: random-effects forest

**Figure 19:**
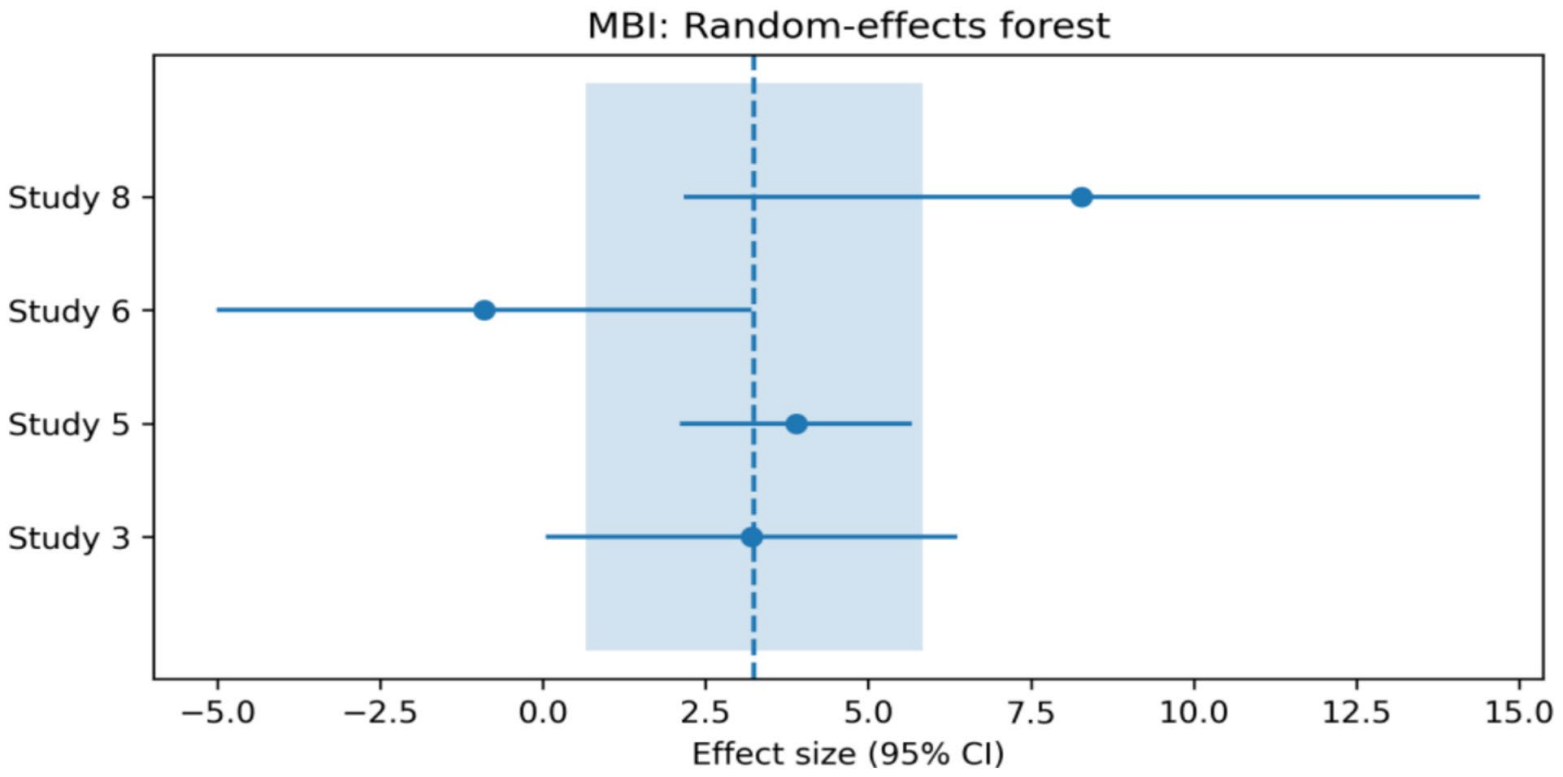
Random-effects forest plot for MBI.

The random-effects forest plot for MBI reveals some heterogeneity, with Study 6 contributing a wide confidence interval (Figure 19).

#### Cumulative random-effects plots

**Figure 20:**
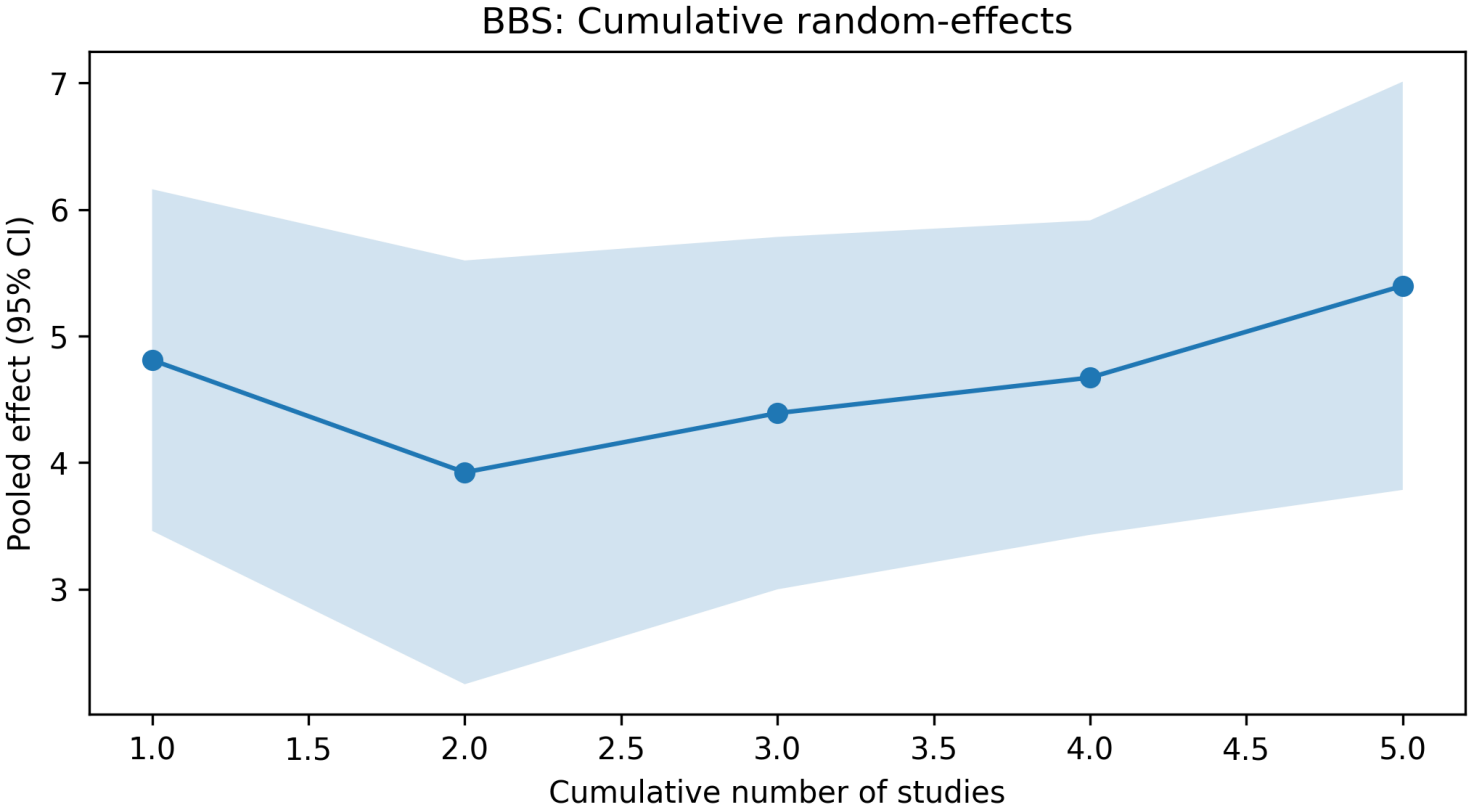
Cumulative random-effects plot for BBS.

The cumulative random-effects plot for BBS demonstrates how the pooled effect changes as more studies are added. This highlights the stability of the pooled estimate as the studies accumulate (Figure 20).

**Figure 21:**
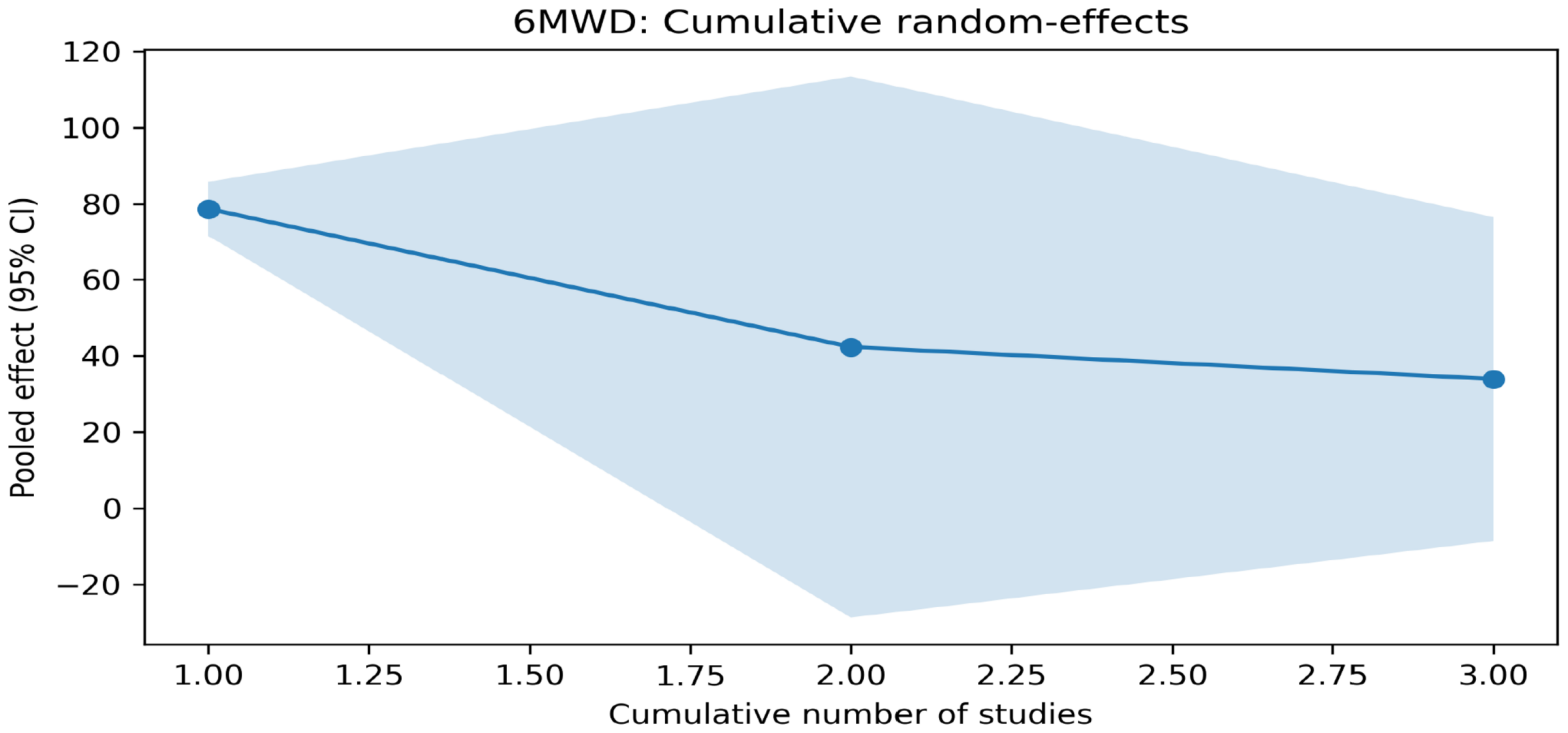
Cumulative random-effects plot for 6MWD.

The cumulative plot for 6MWD shows a gradual decrease in the pooled effect size as more studies are incorporated, indicating a strong influence of the earlier studies (Figure 21).

**Figure 22:**
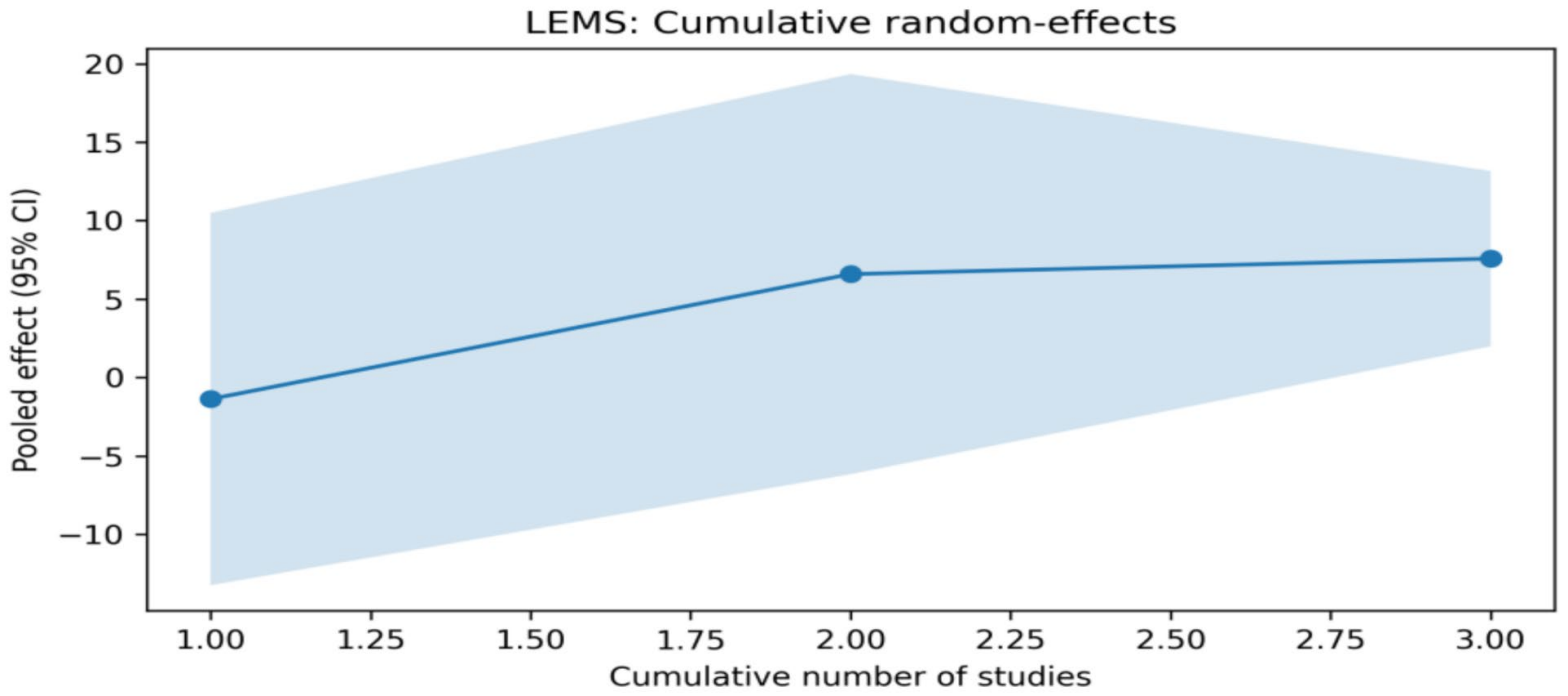
Cumulative random-effects plot for LEMS.

The cumulative plot for LEMS shows a consistent increase in the pooled effect as more studies are included, suggesting a reinforcing effect (Figure 22).

**Figure 23:**
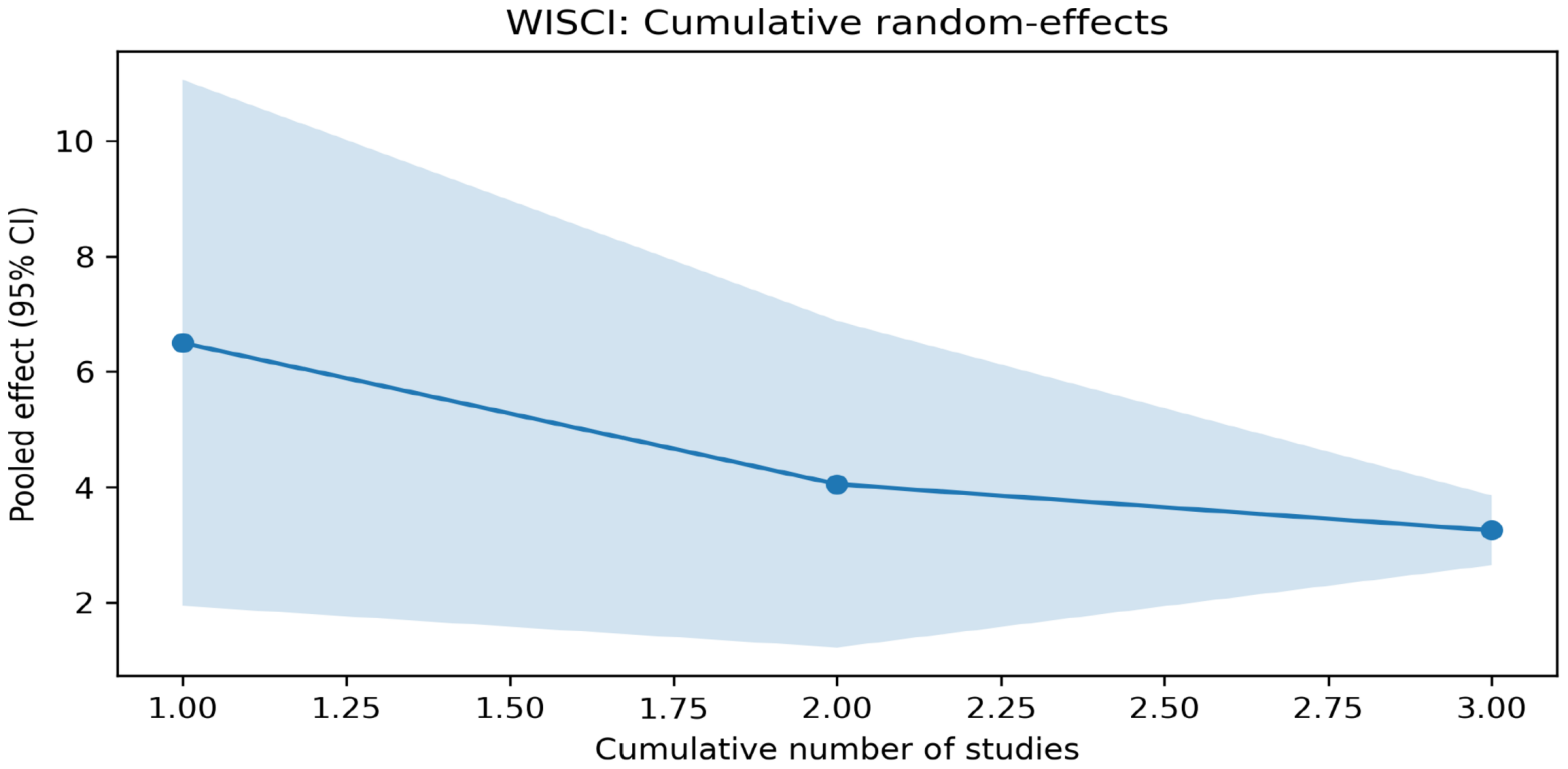
Cumulative random-effects plot for WISCI.

The cumulative plot for WISCI shows minimal change in the pooled effect across studies, indicating a stable estimate (Figure 23).

**Figure 24:**
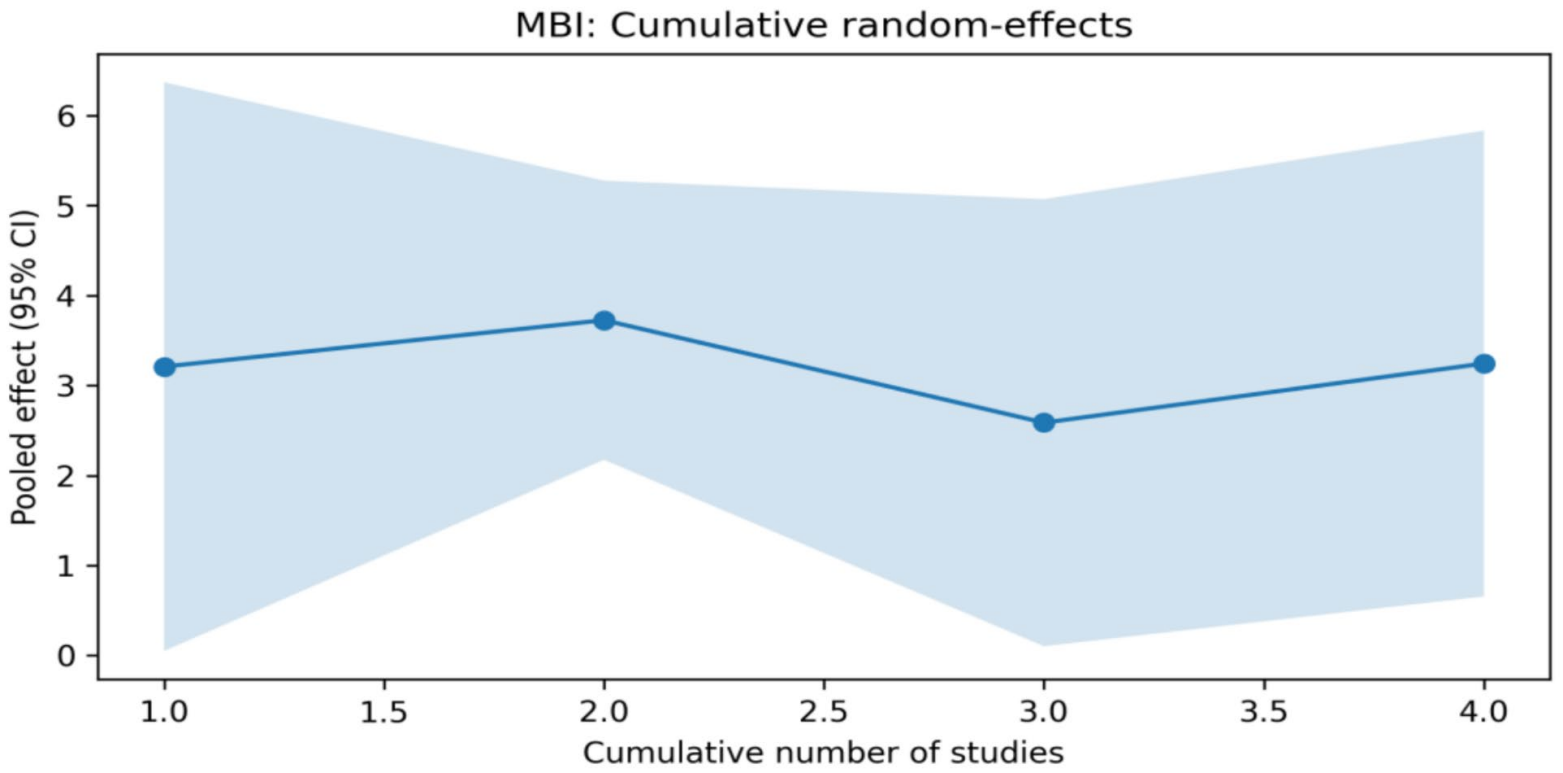
Cumulative random-effects plot for MBI.

The cumulative random-effects plot for MBI shows minimal fluctuations in the pooled effect, with more studies adding stability to the estimate (Figure 24).

**Figure 25:**
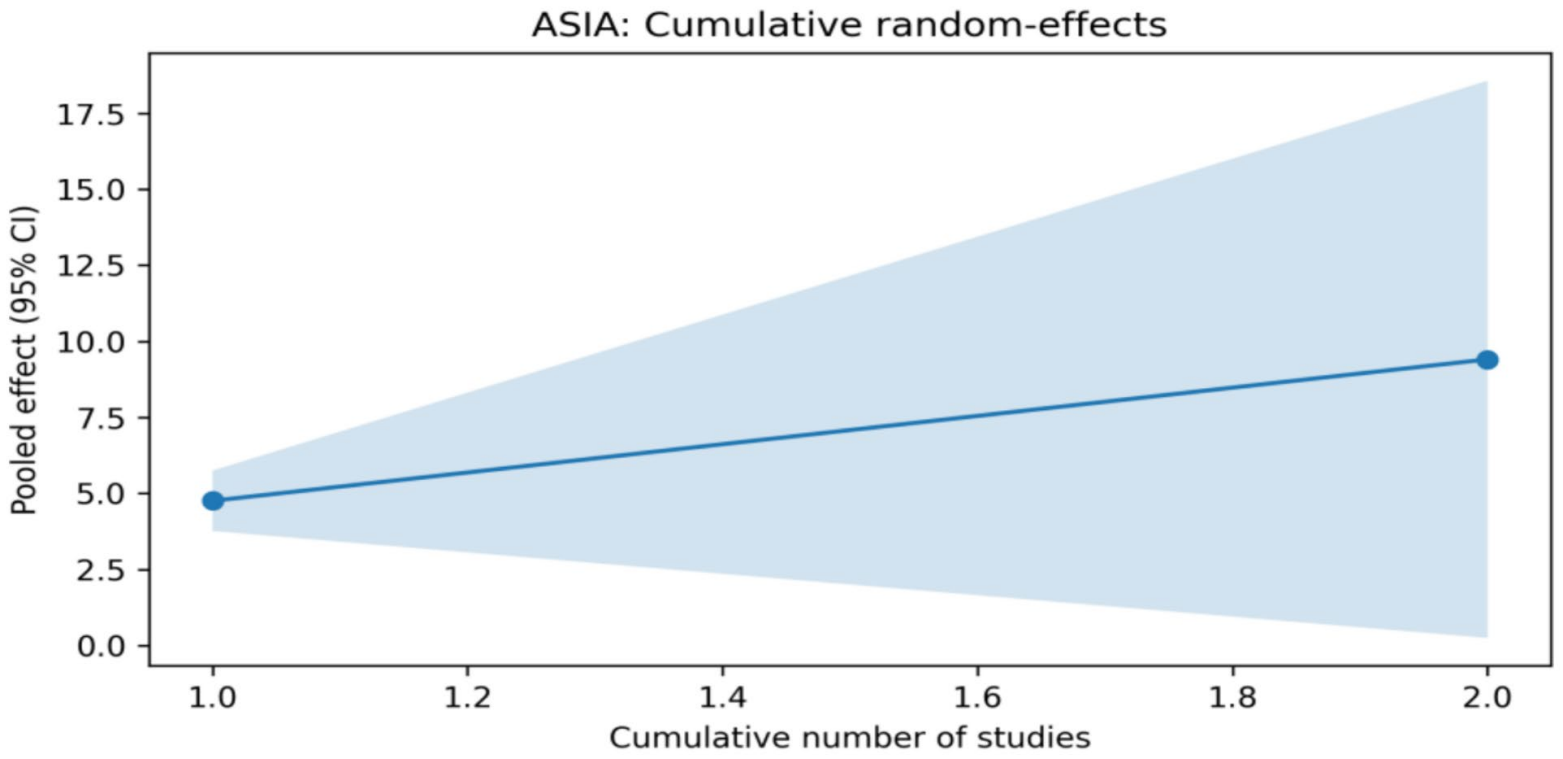
Cumulative random-effects plot for Asia.

The cumulative plot for ASIA shows a gradual increase in the pooled effect, though it is still subject to considerable variation due to the small number of studies (Figure 25).

## Discussion

This study is a systematic review and meta-analysis evaluating that estimated the impact of exoskeleton-assisted rehabilitation on balance, walking performance, strength of motor activity, gait independence, and activities of daily living in patients with spinal cord injury (SCI). The combined findings suggest that the utilization of exoskeletons was related to statistically significant changes in a variety of functional outcomes, such as balance, walking endurance, lower extremity motor scores, WISCI-II and activities of daily living. The reported results are consistent with previous meta-analytic data, indicating that robot-assisted and exoskeleton-aided gait training might have functional advantages compared to traditional rehabilitation alone (Liu et al., 2025; Park et al., 2024). Nonetheless, a high level of heterogeneity in some outcomes, including walking endurance and ASIA motor scores, suggests that the results should be interpreted with caution and that there is variability in the study design, patient characteristics, and intervention protocols.

Compared with previous meta-analyses, including those by Liu et al. (2025) and Park et al. (2024), the present study provides several additional contributions. While earlier reviews primarily focused on robotic-assisted gait training or specific subgroups of patients, our analysis includes a broader range of exoskeleton-assisted interventions and outcome measures, including balance, walking endurance, motor function, and activities of daily living. In addition, this study incorporates more recent evidence and provides a more detailed quantitative synthesis of functional outcomes. These differences allow for a more comprehensive evaluation of the potential benefits of exoskeleton-assisted rehabilitation in individuals with spinal cord injury.

The functional improvements after exoskeleton training can be explained by the conceptual framework according to which intensive and repetitive, task-related training stimulates neuroplasticity and motor learning in neurological injury (He, 2024). Systematic reviews and meta-analyses also reported the improvement of walking ability, strength, and activities of daily living in the studies but the effect on gait speed or gait symmetry was inconsistent across the studies (Park et al., 2024). As an illustration, certain controlled trials have shown that exoskeleton gait training enhanced balance and walking capacity, but other studies have also shown less drastic or no significant improvements of particular gait parameters (Forte et al., 2022; Gupta et al., 2023). Such inconsistencies might indicate variation in the type of devices, length of intervention, chronicity of the patient and outcome measures.

The literature has also covered the safety and acceptability of exoskeleton use. Miller et al. (2016) indicated that powered exoskeletons enable safe ambulation in SCI individuals without any severe adverse incidents, which is consistent with the fact that no severe adverse events have occurred in the present analysis. Users have also expressed positive attitudes and acceptability toward exoskeletons, implying that patients are generally receptive in this technology (Fortin-Bédard et al., 2024). However, the obstacles including cost, accessibility of the device, and standardized training protocols can be still considered as the obstacles to wider clinical adoption.

Although the signs of progress are positive, one must admit several constraints. First, the small sample sizes and the limited number of controlled trials in aggregated results decrease the accuracy of pooled estimates and limit the possibility to assess the formal bias. Second, the variation in intervention attributes and subject groups leads to differences in effect sizes, especially in such outcomes as walking endurance. More conclusive findings would be achieved through research designs that implement standardized protocols and stratify them according to severity and chronicity of the injury. Lastly, there are still to be outlined long-term effects and optimum training parameters.

Overall, exoskeleton-aided rehabilitation seems to have a correlation to significant positive change in various functional areas among persons with SCI in comparison with traditional rehabilitation. These results have been added to the growing body of research showing that robotic gait training technologies have an emerging role in neuro-rehabilitation (Tarnacka, 2023). Further large-scale randomized controlled studies of high quality are required in the future refinement of clinical recommendations and also to determine patient subgroups most likely to respond to exoskeleton-based interventions.

The heterogeneity observed across studies can be attributed to several factors. First, participant characteristics varied substantially, including differences in injury level (cervical vs. thoracic), severity (complete vs. incomplete), and time since injury (acute vs. chronic), all of which influence rehabilitation potential. Second, intervention protocols differed in terms of duration, frequency, and intensity of training, ranging from short-term sessions to multi-week rehabilitation programs. Third, different types of exoskeleton devices were used across studies, each with distinct control strategies and levels of assistance. Finally, variations in outcome measures and assessment time points further contributed to variability in effect sizes. These differences highlight the need for standardized protocols in future research.

The findings of this meta-analysis provide important evidence supporting the role of exoskeleton-assisted rehabilitation as an effective adjunct to conventional therapy in individuals with SCI. The observed improvements across multiple functional domains—including balance, walking endurance, motor function, and independence—suggest that exoskeleton training may facilitate task-specific motor relearning and enhance neuroplasticity. Compared with existing literature, the present study offers a more integrated evaluation of functional outcomes, allowing for a broader understanding of rehabilitation benefits beyond isolated measures. Importantly, while the magnitude of improvements was consistent across outcomes, variability between studies highlights the influence of patient characteristics and intervention protocols, emphasizing the need for individualized rehabilitation strategies.

Taken together, these findings reinforce the growing role of exoskeleton-assisted rehabilitation in neurorehabilitation while highlighting the need for more standardized and targeted clinical applications.

### Clinical implications and future research directions

The findings of this study suggest that exoskeleton-assisted rehabilitation can be considered a valuable adjunct to conventional therapy for improving functional outcomes in individuals with spinal cord injury. Clinically, its use may enhance task-specific gait training, particularly in supervised rehabilitation settings, and may be most beneficial for selected patient populations based on injury characteristics.

Future research should focus on large-scale, high-quality randomized controlled trials with standardized intervention protocols to reduce heterogeneity and improve comparability. Additionally, long-term follow-up studies are needed to assess the sustainability of functional gains, and further research should aim to identify patient subgroups most likely to benefit from exoskeleton-assisted rehabilitation.

## Conclusion

When compared with conventional rehabilitation, exoskeleton-assisted rehabilitation was linked to great balance, endurance of walking, lower-limb motor strength, independence in gait, and activities of daily living. Despite the fact that these results indicate that wearable lower-limb exoskeletons can provide meaningful functional benefits as components of organized rehabilitation programs, the heterogeneity of certain findings is substantial enough to suggest that results must be viewed with caution. The differences in the injuries, types of devices, and the nature of interventions were likely to have led to varying effect sizes in different studies. In general, the evidence at hand indicates that exoskeleton-based training may be used as an addition to the traditional rehabilitation instead of an alternative, and it is necessary to conduct larger and quality randomized controlled trials with standardized procedures to determine how to select patients, what to train, and what the functional outcomes are in the long-term perspective.

## Data Availability

The original contributions presented in the study are included in the article/supplementary material, further inquiries can be directed to the corresponding author.
